# Escitalopram and NHT normalized stress-induced anhedonia and molecular neuroadaptations in a mouse model of depression

**DOI:** 10.1371/journal.pone.0188043

**Published:** 2017-11-15

**Authors:** Or Burstein, Motty Franko, Eyal Gale, Assaf Handelsman, Segev Barak, Shai Motsan, Alon Shamir, Roni Toledano, Omri Simhon, Yafit Hirshler, Gang Chen, Ravid Doron

**Affiliations:** 1 School of Behavioral Science, The Academic College Tel-Aviv-Yaffo, Tel-Aviv, Israel; 2 Department of Education and Psychology, The Open University, Raanana, Israel; 3 School of Psychological Sciences, Tel-Aviv University, Tel-Aviv, Israel; 4 The Sagol School of Neuroscience, Tel-Aviv University, Tel-Aviv, Israel; 5 Faculty of Medicine, Technion–Israel Institute of Technology, Haifa, Israel; 6 Mazor Mental Health Center, Akko, Israel; 7 Center for Translational Systems Biology and Neuroscience, Nanjing University of Chinese Medicine, Nanjing, China; 8 Key Laboratory of Integrative Biomedicine for Brain Diseases, Nanjing University of Chinese Medicine, Nanjing, China; Nathan S Kline Institute, UNITED STATES

## Abstract

Anhedonia is defined as a diminished ability to obtain pleasure from otherwise positive stimuli. Anxiety and mood disorders have been previously associated with dysregulation of the reward system, with anhedonia as a core element of major depressive disorder (MDD). The aim of the present study was to investigate whether stress-induced anhedonia could be prevented by treatments with escitalopram or novel herbal treatment (NHT) in an animal model of depression. Unpredictable chronic mild stress (UCMS) was administered for 4 weeks on ICR outbred mice. Following stress exposure, animals were randomly assigned to pharmacological treatment groups (i.e., saline, escitalopram or NHT). Treatments were delivered for 3 weeks. Hedonic tone was examined via ethanol and sucrose preferences. Biological indices pertinent to MDD and anhedonia were assessed: namely, hippocampal brain-derived neurotrophic factor (BDNF) and striatal dopamine receptor D2 (*Drd2*) mRNA expression levels. The results indicate that the UCMS-induced reductions in ethanol or sucrose preferences were normalized by escitalopram or NHT. This implies a resemblance between sucrose and ethanol in their hedonic-eliciting property. On a neurobiological aspect, UCMS-induced reduction in hippocampal BDNF levels was normalized by escitalopram or NHT, while UCMS-induced reduction in striatal *Drd2* mRNA levels was normalized solely by NHT. The results accentuate the association of stress and anhedonia, and pinpoint a distinct effect for NHT on striatal *Drd2* expression.

## Introduction

Since the time of Epicurus [1], an ancient Greek philosopher, pleasure has been stipulated as a vital ingredient of human well-being. DSM-V [2] defines anhedonia as the diminished ability to obtain pleasure from otherwise positive stimuli and as a keystone symptom of various neuropsychiatric disorders, such as major depressive disorder (MDD). Pizzagali [3] proceeds further and solicits anhedonia as one of the most promising endophenotypes of MDD. The current treatise aims to further examine the relationship between hedonic faculty and bio-behavioral state.

As remission rate following selective serotonin reuptake inhibitors (SSRIs) treatment for MDD is roughly 45% [4,5] and SSRIs treatment is associated with frequent adverse effects, such as orgasm dysfunction in as up to 37% of the patients [4], it is of utmost importance to develop new efficacious pharmacotherapies that also mitigate the reward-related adverse effects of SSRIs. In previous studies from our lab we demonstrated a therapeutic effect of a novel herbal treatment (NHT) in reducing depressive- and anxiety-like behaviors in the unpredictable chronic mild stress (UCMS) animal model of depression. Specifically, we showed how chronic NHT administration prevented the UCMS-induced increments in time of immobility in the forced swim test (FST), passive coping in the tail suspension test (TST) and anxiety-like behavior in the elevated plus maze (EPM) [6,7]. Moreover, we demonstrated how NHT had no negative effect on sexual function in mice, in contrast to the SSRI escitalopram [6]. The effect of NHT on UCMS-induced anhedonia was not examined yet.

Alcohol is perceived by consumers as a pleasurable substance of choice [8]. The consumption of alcohol promotes activity of the brain reward system (BRS), as depicted by dopamine secretion in the nucleus accumbens (NAc) in both rodents [9] and humans [10]. The BRS has an important functional role by regulating hedonic state, motivation, decision-making and learning processes [11]. The inability to attain hedonic state in an adequate manner is characteristic of destabilized BRS [12] and is correlated with poorer well-being as expressed in self-report surveys [13] and underlain by dopaminergic alterations [14]. Most individuals consume alcohol for recreational purposes, as merely 15.4% of all alcohol users were reported with alcohol dependence [15]. The transition from controlled to maladaptive alcohol or other drug use is an intricate process affected by varying genetic and environmental factors. The neurobiological and molecular implications of chronic, addiction-related versus acute or sub-chronic, moderate drug use differ significantly, thus stating that ample portion of alcohol use has no negative implications on the BRS, and might even imply normative hedonic-prompting behavior [16].

Ethanol has been widely employed in animal models of addiction [17], but is not frequently applied in models screening hedonic tone. The present design aims to place under scrutiny the possible utilization of ethanol preference test as a pre-clinical instrument for testing the reward system. Our conjecture was that moderate ethanol preference is an indicator of regulated hedonic quality. We conducted an experiment in which ethanol preference was obtained following stress exposure and pharmacological treatments. The applied drugs were the SSRI escitalopram and NHT. Consequently, a second experiment was designed to study the effects of stress and escitalopram / NHT treatments on the hedonic tone prompted by sucrose, a primary reinforcer vastly used in animal models of anhedonia [18–20]. This design makes it feasible to discriminate between the rewarding potencies of the two substances (ethanol and sucrose) under naïve and stress conditions, with or without the aforementioned treatments.

Mice were exposed to UCMS for 4 weeks, treated with escitalopram, NHT or vehicle for 3 weeks, and then screened for hedonic tone and pertinent neurobiological markers. We examined whether NHT will attenuate the UCMS-induced anhedonia and whether NHT has a specific effect on an important factor of the BRS, i.e., dopamine receptor D2 (*Drd2*) gene expression in the striatum. We additionally assessed hippocampal brain-derived-neurotrophic-factor (BDNF) to confirm our prior finding that UCMS-induced down-regulation in BDNF levels can be averted by NHT [7]. The behavioral and neuromolecular effects of NHT were compared with those of escitalopram.

## Materials and methods

### Animals

One-month-old ICR outbred male mice (Envigo, Israel) were kept in the vivarium of the ‘Academic College of Tel-Aviv-Yaffo’. Mice were housed in standard group cages (5 mice per cage, each cage containing mice from all experimental groups) and kept on a reversed 12 h light/dark cycle (light on 19:00–7:00). Mice had *ad*-*libitum* access to food and water except during stressor application (with the exclusion of the light/dark cycle reversal). All experiments were carried out in strict accordance with the recommendations in *The Guide for the Care and Use of Laboratory Animals of the National Institutes of Health* and were approved by the Institutional Animal Care and Use Committee of the 'Academic College of Tel-Aviv-Yaffo' (*Permit Number*: *mta-2015-09-5*). Animal sacrifice was executed via cervical dislocation by an experienced experimenter. All efforts were made to minimize animal suffering.

The ICR outbred mouse is a species that entails high genetic variability between animals, and therefore has a relatively better ecological validity compared to other transgenic mice and was utilized for this study [21].

### UCMS

The procedure is grounded on the paradigm originally designed for rats by Katz [22] and subsequently Willner [23]. It was previously adapted to mice, whilst applying an unpredictable stressor regime [24]. The following stressors were applied: cages with 1 cm of water at the bottom (water stress), inversed light/dark cycle, cages with wet sawdust, tilted cages at 45 degrees, mice restrain, empty cages and cages with the sawdust of different mice. A single stressor was applied for 4 h daily, during a period of 4 weeks. Contrastingly, the light/dark cycle disruption was applied from mid-day Friday until Sunday morning. To prevent habituation and to provide an unpredictable feature, stressors' schedules were altered daily.

### Drugs

NHT is composed of Crataegus Pinnatifida, Triticum Aestivu, Lilium Brownie and Fructus Zizyphi Jujubae. Herbs were purchased as freeze-dried granules from KPC Products Inc. (Irvine, CA, USA). NHT was prepared by dissolving the 4 herbs (together) in saline, containing 1% DMSO to give a final concentration of 0.47 mg/ml (each). NHT was administered daily (30 mg/kg; i.p.). The dose was opted based on our previous study [6].

Escitalopram was kindly donated by TEVA Pharmaceutical Industries Ltd. and was administered daily (15 mg/kg; i.p.). The dose was opted based on previous studies [25,26].

Saline was administered at a weighed dose of 1% of the mice current weight (i.p.).

### Behavioral assessment: Two bottle choice (sucrose/ethanol)

After the treatment phase, mice were single housed for a period of 6 days. Two drinking nozzles were set at the cage through which the animal could intake distilled water and either a 10% ethanol solution (experiment 1) or a 2% sucrose solution (experiment 2). The nozzles' positions were switched after 3 days to counterbalance the effect of position preference, in acquiescence with previous reports[19]. Fresh fluids were supplied after bottles' weight assessments. Sucrose and ethanol solutions were introduced for the first time during the assessment period, and there were neither prior acclimation nor habituation phases. Six-day preference was calculated per mouse as ratio of sucrose or ethanol mean intake from total fluid mean intake (i.e., ethanol or sucrose / ethanol or sucrose + water).

### Assessment of BDNF levels

Mice' brains were removed and rinsed of blood after sacrifice and the hippocampus and striatum were dissected out entirely. Tissues were homogenized in a cold extraction buffer (Tris-buffered saline, pH 8.0, with 1% NP-40, 10% glycerol, 5 mM NaMetavanadate, 10 mM PMSF, 100 μg/ml aprotinin and 10 μg/ml leupeptin). Homogenates were acidified with 0.1 M HCl (pH 3.0), incubated at room temperature (22–24°C) for 15 min, and neutralized with 0.1 M NaOH (pH 7.6). Homogenates were then microfuged at 7,000 g for 10 min. BDNF levels were evaluated using sandwich enzyme-linked immunosorbent assay (ELISA) as previously described [27]. BDNF concentrations are presented after normalization to total protein levels.

### Assessment of *Drd2* mRNA expression levels

Quantitative reverse transcriptase polymerase chain reaction (qRT-PCR) was conducted as previously described [28]. Briefly, RNA was executed with TRIzol reagent and precipitated with 100% ethanol and 0.3 M NaAcetate. mRNA was reverse transcribed with RevertAid cDNA synthesis kit. Expression was quantified via quantitative real time PCR (StepOnePlus: Applied Biosystems, Foster City, CA, USA) using the ∆∆Ct method. We used the following primers to amplify specific cDNA regions: *Drd2*, forward 5'-GACACCACTCAAGGGCAACT-3'; reverse 5'-TCCATTCTCCGCCTGCCTGTTCAC-3'; Gapdh, forward 5'-GCAAGAGAGAGGCCCTCAG-3'; reverse 5'-TGTGAGGGAGATGCTCAGTG-3'.

### Study design

#### Experiment 1—ethanol

**Fig 1:**
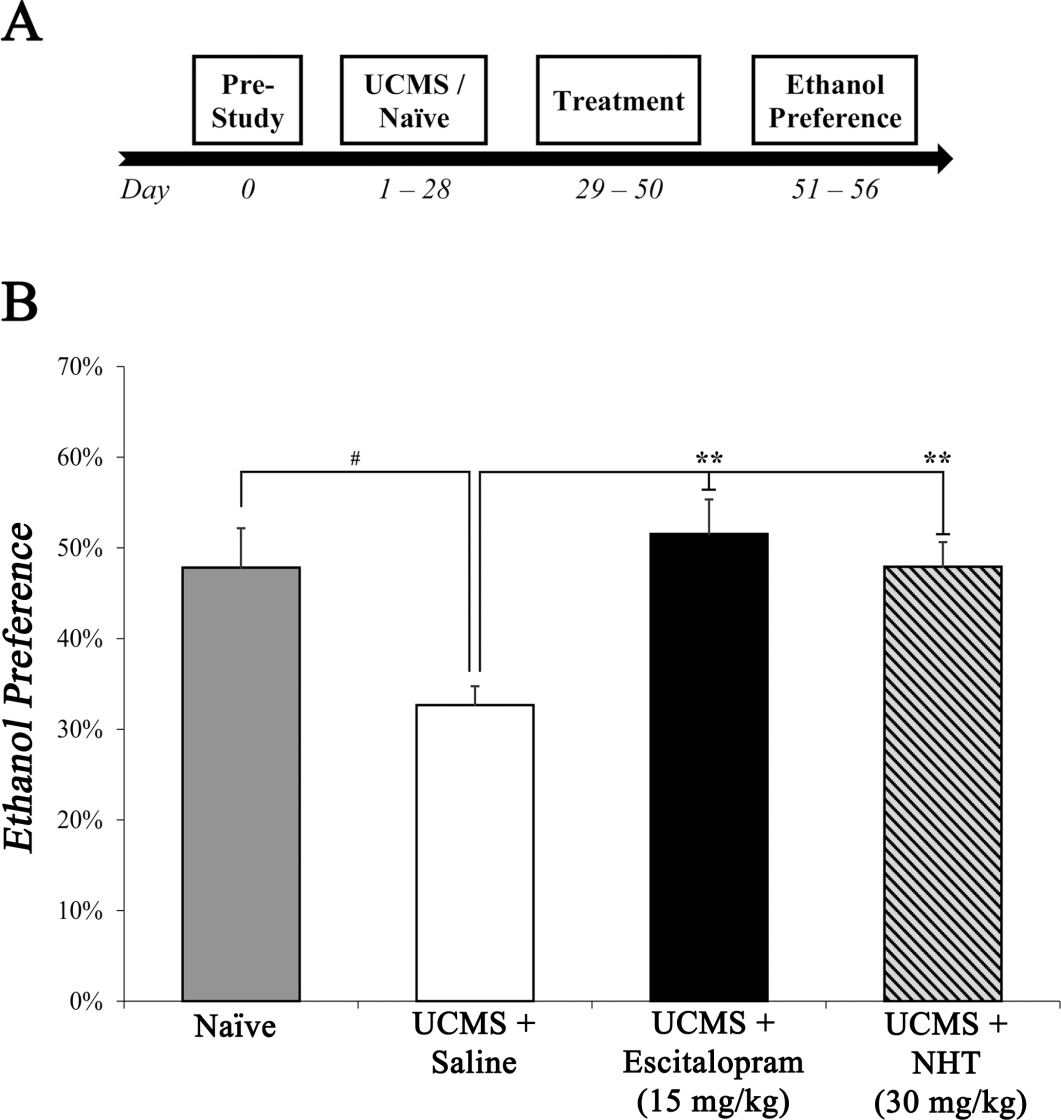
The effect of escitalopram (15 mg/kg) and NHT (30 mg/kg) treatments on stress-induced alterations in ethanol preference. (A) A diagram depicting study design of experiment 1. After acclimation, mice were submitted to UCMS or naïve conditions (4 weeks), subsequently treated with saline, escitalopram or NHT (3 weeks) and screened for ethanol preference (6 days). (B) Stress diminished ethanol preference, while both NHT and escitalopram reversed this stress-induced diminution. *n* = 15–17 mice per group. ^#^*P*<0.05 vs. naïve group. **P<0.01 vs. UCMS + saline group.

UCMS procedure was administered on ICR outbred mice. Escitalopram or NHT, were injected for 3 weeks following stress protocol, balanced with saline-injected mice and home cage naïve controls. Thereafter, mice were subjected to two-bottle-choice procedure in which they were tested for their ethanol preference (see Fig 1A for study design).

#### Experiment 2 –sucrose

**Fig 2:**
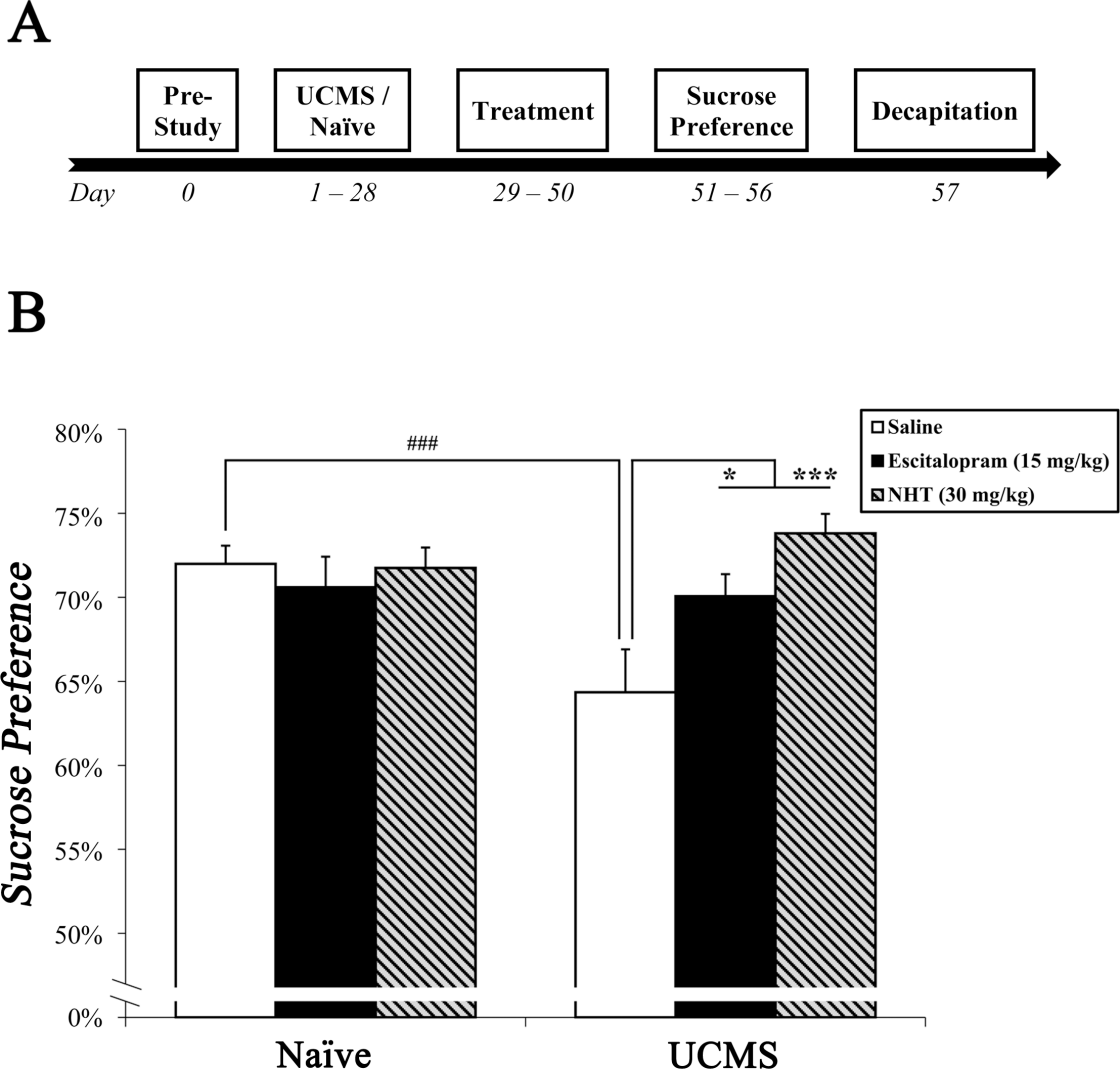
The effect of escitalopram (15 mg/kg) and NHT (30 mg/kg) treatments on stress-induced alterations in sucrose preference. (A) A diagram depicting study design of experiment 2. After acclimation, mice were submitted to UCMS or naïve conditions (4 weeks), subsequently treated with saline, escitalopram or NHT (3 weeks), screened for sucrose preference (6 days) and prepared for neurobiological assessments. (B) Stress diminished sucrose preference, while both NHT and escitalopram reversed this stress-induced diminution. *n* = 15–17 mice per group. ^###^*P*<0.001 vs. naïve + saline group. *P<0.05, ***P<0.001 vs. UCMS + saline group.

Mice were subjected to UCMS or remained non-stressed (naïve). Stressed and naïve mice were then treated with escitalopram, NHT or saline for a period of 3 weeks and subsequently underwent the sucrose preference test (see Fig 2A for study design). Shortly after, mice were sacrificed, and their brains were removed. Biological indices pertinent to MDD and anhedonia were assessed: namely, hippocampal BDNF levels and striatal *Drd2* mRNA levels.

### Data analysis and interpretation of results

Results are expressed as mean +/- SEM. In the ethanol experiment, data was analyzed using one-way ANOVA. Other data was analyzed using two-way ANOVA with pharmacological treatment and stress manipulation as between subject variables. ANOVA was followed by Sidak post-hoc analysis. Significance was assumed as *P*<0.05.

## Results

### Ethanol preference

#### NHT and escitalopram normalized the stress-induced reduction in ethanol preference

One-way ANOVA revealed a significant main effect for treatment (*F*_(3,61)_ = 6.785, *P*<0.001; Fig 1B). Sidak post-hoc analysis revealed that saline-treated stressed mice exhibited lower ethanol preference compared to naïve mice (non-stressed, non-treated) (*P*<0.05). In Addition, escitalopram- and NHT-treated stressed mice showed significantly higher ethanol preference compared to saline-treated stressed mice (*P*<0.01 in both contrasts). No significant differences were found between the escitalopram or NHT groups to the naïve group (*N*.*S*.).

### Sucrose preference

#### NHT and escitalopram normalized the stress-induced reduction in sucrose preference

Two-way ANOVA revealed a significant manipulation × treatment interaction (*F*_(2,92)_ = 4.917, *P*<0.01; Fig 2B). Saline-treated stressed mice exhibited lower sucrose preference compared to saline-treated naïve mice (post-hoc: *P*<0.001). In addition, escitalopram- and NHT-treated stressed mice exhibited higher sucrose preference compared to saline-treated stressed mice (post-hoc: *P*<0.05 and *P*<0.001, respectively), which was analogous to the sucrose preference demonstrated by naïve mice (*N*.*S*.).

### Hippocampal BDNF levels

#### NHT and escitalopram normalized the stress-induced reduction in BDNF levels

**Fig 3:**
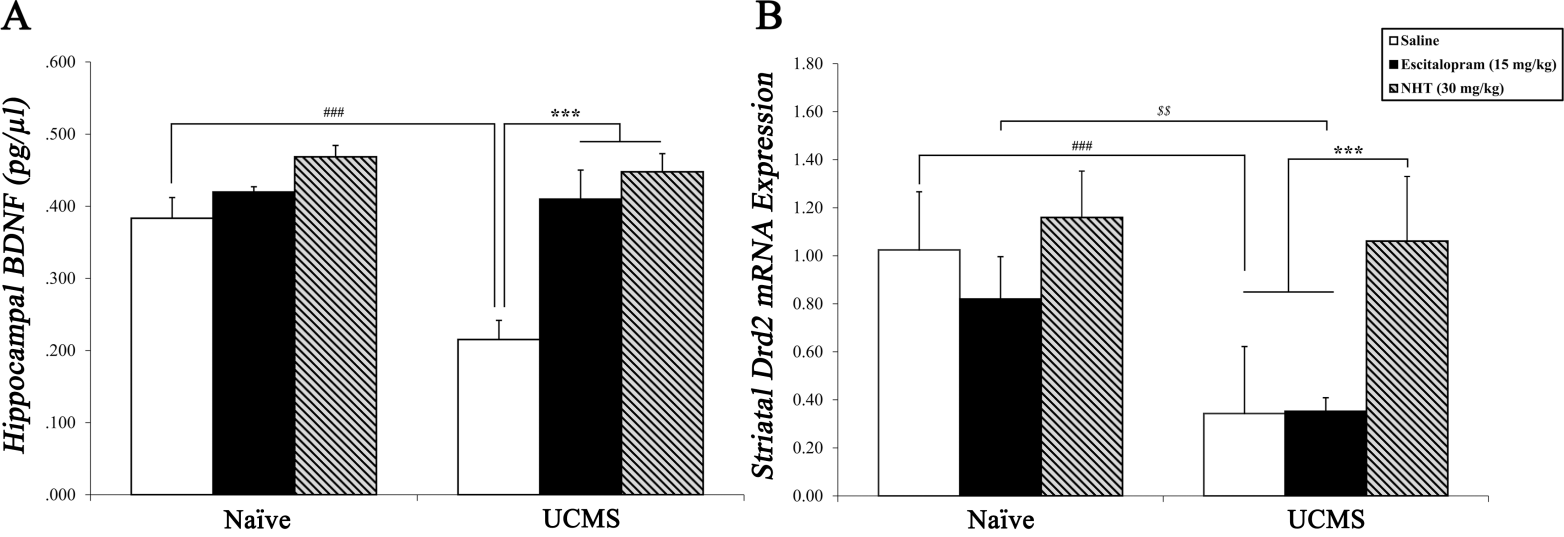
The effects of escitalopram (15 mg/kg) and NHT (30 mg/kg) treatments on stress-induced alterations in hippocampal BDNF and striatal *Drd2* levels. (A) Stress reduced hippocampal BDNF concentration, while both NHT and escitalopram normalized this stress-induced reduction. *n* = 4–5 mice per group. ^###^*P*<0.001 vs. naïve + saline group. ***P<0.001 vs. UCMS + saline group. (B) Stress reduced striatal *Drd2* mRNA expression under both saline and escitalopram treatments, but not under NHT treatment. *n* = 4–6 mice per group. ^###^*P*<0.001 vs. naïve + saline group. ^*$ $*^*P*<0.01 vs. naïve + escitalopram group. ***P<0.001 vs. UCMS + NHT group.

Two-way ANOVA revealed a significant manipulation × treatment interaction (*F*_(2,22)_ = 5.188, *P*<0.05; Fig 3A). Saline-treated stressed mice exhibited lower BDNF levels compared to saline-treated naïve mice (post-hoc: *P*<0.001). In addition, escitalopram and NHT-treated mice showed higher BDNF levels compared to saline-treated stressed mice (post-hoc: *P*<0.001 in both contrasts). No significant differences in BDNF levels were found between the escitalopram-, NHT- and naïve-saline groups (*N*.*S*.).

### Striatal *Drd2* mRNA levels

#### NHT averted the stress-induced down-regulation in *Drd2* mRNA levels, as opposed to escitalopram

Two-way ANOVA revealed a significant manipulation × treatment interaction (*F*_(2,23)_ = 4.522, *P*<0.05; Fig 3B). Saline-treated stressed mice exhibited lower striatal *Drd2* levels compared to saline-treated naïve mice (post-hoc: *P*<0.001); similar stress-induced down-regulation was found among escitalopram-treated mice (post-hoc: *P*<0.01). Unlike escitalopram-treated stressed mice, NHT-treated stressed mice showed significantly higher levels of striatal *Drd2* compared to saline-treated stressed mice (post-hoc: *P*<0.001).

## Discussion

The present study explored the hedonic tone in an animal model of depression and the effects of escitalopram and NHT. It yielded several important findings: [1] UCMS reduced ethanol/sucrose preferences and escitalopram or NHT restored baseline preference. In our study ethanol played a parallel role to sucrose, suggesting that in ICR mice ethanol consumption could function as an immediate reinforcer, instigating the reward system; [2] the behavioral outcome was supplemented by neurobiological alteration, viz. restoration of UCMS-induced diminution in hippocampal BDNF levels found in both escitalopram- and NHT-treated mice; and [3] striatal *Drd2* mRNA levels were reduced by stress manipulation. NHT had an enhancing and balancing effect on *Drd2* expression, whilst escitalopram did not.

All the presented biochemical data was obtained in the sucrose experiment. We did not apply those tests in the ethanol group, since previous studies reported on upregulation in *Bdnf* expression after acute ethanol consumption [29], which might have confounded the results. The same logic was applied to the *Drd2* assessments following the datum that ethanol exposure yields significant alterations in DRD2 expression [30]. Data was obtained through the sucrose group, where a precise delineation was more attainable. The dopaminergic reaction in the NAc to sucrose consumption wanes rapidly, and has no effect in the following tests [31]. This is in contrast to the robust dopaminergic alterations exhibited after exposure to various drugs of abuse, including ethanol [32,33].

The validity of UCMS as a construct reflecting the pathogenesis, bio-symptomatology and phenomenology of depression in humans has been frequently debated [34], being chiefly accepted as a valid model for pharmacological screenings and neurobiological examinations reminiscing mood and anxiety disorders [35]. One of the most bolstering features of UCMS pertaining to this debate is the elicitation of anhedonia [36], a phenomena intrinsically twined with MDD. Loas [37] suggested a model centered on anhedonia in the etiology of depression. His model emphasized that minor anhedonic tone during childhood, also due to exposure to environmental stressors [38,39], is a strong predictor of anterior formation of MDD. Within the realm of MDD, anhedonia was found to be a core domain, stressed by evidence of poorer treatment outcome and more severe depressive symptoms for MDD patients with prominent anhedonia [40,41]. The current results revealed a strong yoke between environmental stress and anhedonia in mice. Nonetheless, the current design does not fully discriminate between anhedonia and other domains of MDD. Such discrimination could be proven fruitful in future research.

In congruence with our previous findings [6,42], a significant diminution of hippocampal BDNF concentration was observed following stress manipulation. Vast literature affirms and typifies the eminent role of BDNF in the etiology of human depression [43,44]. Diminished hippocampal BDNF expression was found in untreated depressive human patients [45] and in stress induction animal models [46]. The 'neurotrophin hypothesis' suggests that BDNF is a gene of utmost importance in the etiology of MDD [47]. Contradictory to the initial 'neurotrophin hypothesis', studies have shown that BDNF plays a diverse role in different brain systems. In the hippocampus and hypothalamus-pituitary-adrenal (HPA) stress-related pathways, lowered BDNF levels are indicators of dysregulation, as exhibited in MDD. In contrast, in the ventral-tegmental area and NAc, reward-related pathways, ascended BDNF expression is signaling imbalance in affect [48,49]. Chronic stress led to dendritic atrophy in the hippocampus and pre-frontal cortex (PFC), and to impaired long-term-potentiation induction in the hippocampal-PFC circuitry [50,51]. In the NAc, on the other hand, chronic stress led to dendritic hypertrophy [52]. The neurobiology of MDD involves deficiencies in both the stress and reward systems; the presented BDNF results convey reinforcement to the established HPA-dysregulation hypothesis of MDD in its relation to BDNF expression.

Although the links between depression, stress, antidepressants and BDNF are well recognized, the mechanisms underlying their interactions are still not soundly established. It is recognized that serotonin and BDNF exert bidirectional (rather than unidirectional) influence, promoting signaling and gene expression of each other [53]. One conjecture regarding the mechanism of this influence suggests that chronic SSRIs treatment (as opposed to stress) facilitates the expression and synthesis of BDNF in hippocampal astrocytic cells; thereby, eliciting neuro-protective and anti-depressive effects [54,55]. Other postulations have suggested that SSRIs can alter phosphorylation of CREB (a transcription factor which is a catalyst of BDNF synthesis) in pertinent signaling pathways; thus, promoting BDNF expression [53,56]. Insight is lacking vis-à-vis the mechanism by which NHT affects BDNF expression. In a previous study [6] we found that NHT upregulated serotonin transporter (SERT) expression in the hypothalamus. Both the hypothalamus and the hippocampus are involved in the inhibitory feedback of the HPA-axis, and are implicated in affective disorders [57,58]. Hence, further studies should be aimed to elucidate whether the effect of NHT on hippocampal BDNF expression is obtained through serotonergic alterations in stress regulatory structures or through other neural mechanisms.

In our study NHT had a balancing effect on *Drd2* expression in the striatum following stress, while escitalopram did not. Previous works reported that pharmacologically induced DRD2 blockaded rats showed a reduced tendency to work for sucrose [59] and chronic mild stress caused a decrease in striatal *Drd2* expression [60,61]. In addition, social isolation of Flinders Sensitive Line rats, genetic model of depression [62], reduced *Drd2* expression in several areas in the striatum [63]. Contrastingly, Zhang et al. [64] reported that chronic unpredictable stress in rats up-regulated *Drd2* mRNA expression in the striatum, with no effect for escitalopram administration compared to saline. Nonetheless, the stress paradigm they applied comprises more severe stressors (e.g., electric footshocks), therefore, might elicit other reactions than the mild stress paradigm we applied. The rational for utilizing mild stressors is that they are more resembling of the pathogenic environmental factor in MDD formation [65], as opposed to severe stress resemblance to trauma-related pathologies.

Neuroimaging studies have illustrated an abnormal activity in several BRS areas of MDD patients, among them the striatum [66,67]. Additionally, the ventral striatum was clinically supported as an effective region for deep brain stimulation (DBS) treatment of refractory depression [68,69]. Studies have indicated a significant involvement of DRD2 in the BRS dysregulation affecting MDD patients, though there are incongruous findings regarding the nature of this involvement [14]. One of the suggested models postulates that recurring activation of the HPA-axis, accompanied by increased secretion of glucocorticoids from the adrenal gland, results in sensitization of the dopaminergic-mesolimbic system. Such hypercortisolemia alters dopamine binding and DRD2 availability in the striatum, which might underlie the changes in hedonic reactivity [70,71]. DRD2 plays an important role in the motivational facet of the reward system [72]; clinical studies found that striatal DRD2 upregulation is a sign of MDD treatment responsiveness to SSRIs [73,74]. Moreover, the use of the DRD2/3 antagonist, sulpiride, annulled the antidepressant effect of SSRI treatment in MDD patients [75]. Deducing from the stated findings it has been hypothesized that sensitization of DRD2 in mesolimbic terminal regions is one of the central mechanisms by which SSRIs exert their therapeutic action [75,76]. Divergent to the aforementioned studies that utilized paroxetine or fluoxetine, escitalopram did not sustain striatal *Drd2* expression following stress in our study. A putative explanation to this finding is that escitalopram is an SSRI with relatively lower affinity to dopamine transporter (DAT) [77]. The mechanism by which NHT altered striatal *Drd2* expression is still unclear and remains to be further examined in future neurobiological and molecular studies. Such attempts are currently being conducted in our lab, in which we aim to identify specific active ingredients of NHT and their pharmacodynamics and biomolecular interactions.

One of the main adverse effects of SSRIs is sexual dysfunction [78]. Dopamine has a focal function in the regulation of sexual behavior [79]. Drugs that enhance dopamine transmission cultivate sexual activity. On the pharmacological aspect, the antidepressant that impairs sexual function the least is bupropion which is a dopaminergic agonist (apart from its effects on norepinephrine and serotonin) [80]. In a previous study we demonstrated that treatment with escitalopram reduced the sexual behavior of mice in comparison with NHT treatment, which had no such negative effect [6]. Our current results may suggest that this difference is underlain by a discrete effect of NHT on the dopaminergic system. The sustainment of striatal *Drd2* levels held by NHT treatment might interact with reactivity to dopamine in the BRS by the receptors, a mechanism that might putatively explain the differences in sexual activity. This distinction could prove fertile in the development of antidepressant medicine free of the frequent adverse effect of sexual dysfunction. However, such presupposition could only be viewed as an initial hypothesis that remains to be examined and corroborated with further pre-clinical and clinical data. Moreover, the *Drd2* stated effect did not reflect per se the behavioral hedonic tone observed in the solution preference tests, where both drugs yielded hedonic effects. This implies that the some aspects of hedonic behavior depend on striatal *Drd2* expression, while others might not.

A resemblance between the patterns of sucrose and ethanol preferences in ICR mice was observed. Some addiction researchers maintain the presupposition that rodents have a natural tendency to avoid alcohol or consume it in an unsatisfactory manner [81]. It is perhaps so when the task at hand is manipulating substance-dependency, with substantial voluntary drug self-administration as an important component in the model's validity [82]. This obstacle seems to ebb in the case of hedonic-related consumption. Numerous animal species display a significant ethanol intake in naïve voluntary conditions including differing inbred mice species [83], rats [84] and primates [85]. In rats, this voluntary consumption phenomena is insufficient to elicit abuse-like phenotype without induction of intermittent withdrawals [86]. Other studies exploring the properties of ethanol in animals found a diminution in ethanol preference in deficient DRD2 mice [87] and a reduction in ethanol preference of rats subdued to chronic mild stress [88,89]. The inferred resemblance we found between ethanol and sucrose preferences highlights the possibility of operationalizing ethanol preference test not solely for addiction research but also for experiments concerning hedonic tone. The use of ICR outbred mice strengthens the suggested notion, considering the 'genetically-based-noise' they entail in their between-animal DNA variability.

## Conclusions

The current research has emphasized the relation between stress and anhedonia, and pinpointed the possible involvement of striatal *Drd2* and hippocampal BDNF levels in their association. Ethanol was shown as a substance eliciting reward behavior, implying the possibility of utilization of ethanol in reward-related experiments and not merely in animal addiction models. Stress had a lessening effect on mice sucrose and ethanol preferences. These effects were reversed via two pharmacotherapies (escitalopram and NHT). Both therapies prevented the down-regulation in hippocampal BDNF levels but bared a distinct impact on striatal *Drd2* expression.
